# Supplementary figures and images for: Small molecule therapeutics for COVID-19: repurposing of inhaled furosemide
Source: PeerJ. 2020 Jul 7;8:e9533. doi: 10.7717/peerj.9533 (PMC7350920; doi:10.7717/peerj.9533)

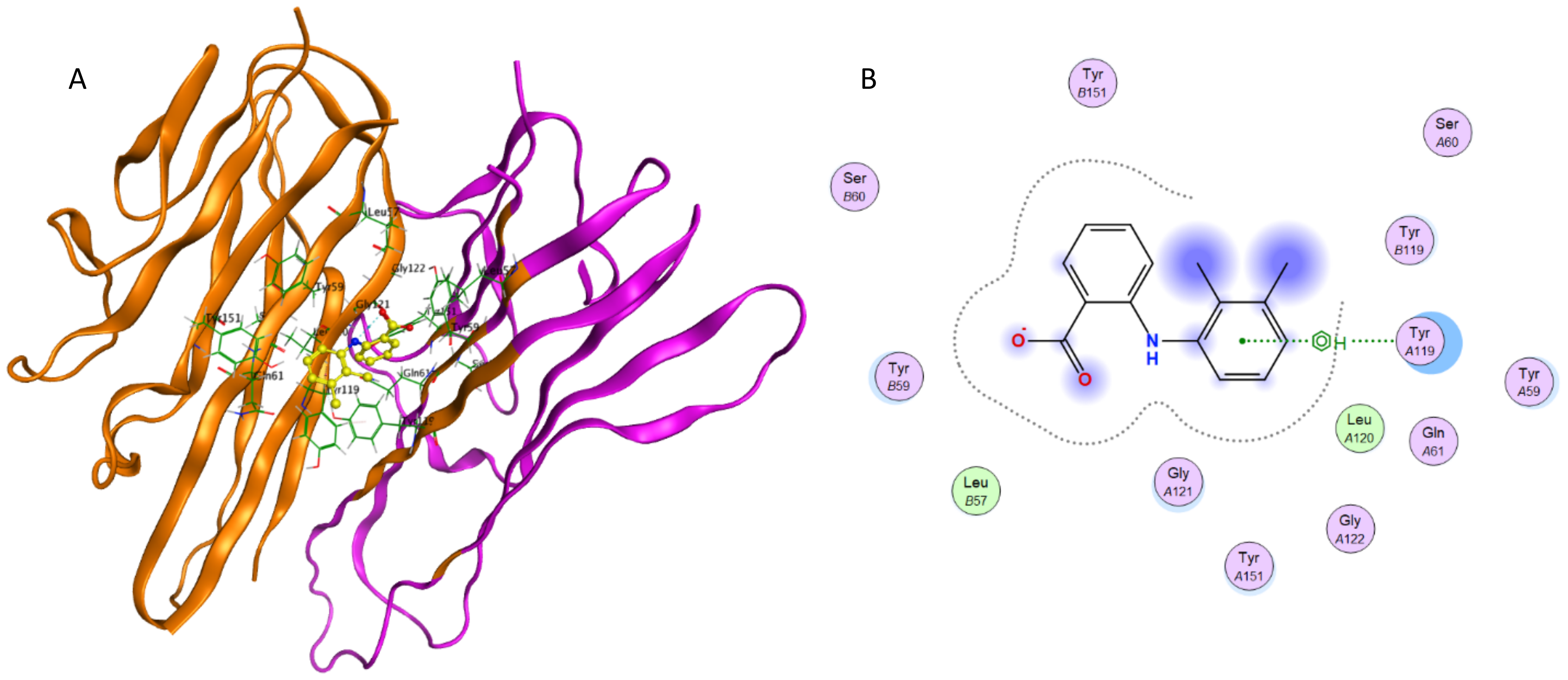

Supplement: Supplemental Information 1 — (A) Binding of mefenamic acid in the active site of TNF-α; (A) Ligand interaction diagram of mefenamic acid in binding site of TNF-α. [file peerj-08-9533-s001.png]

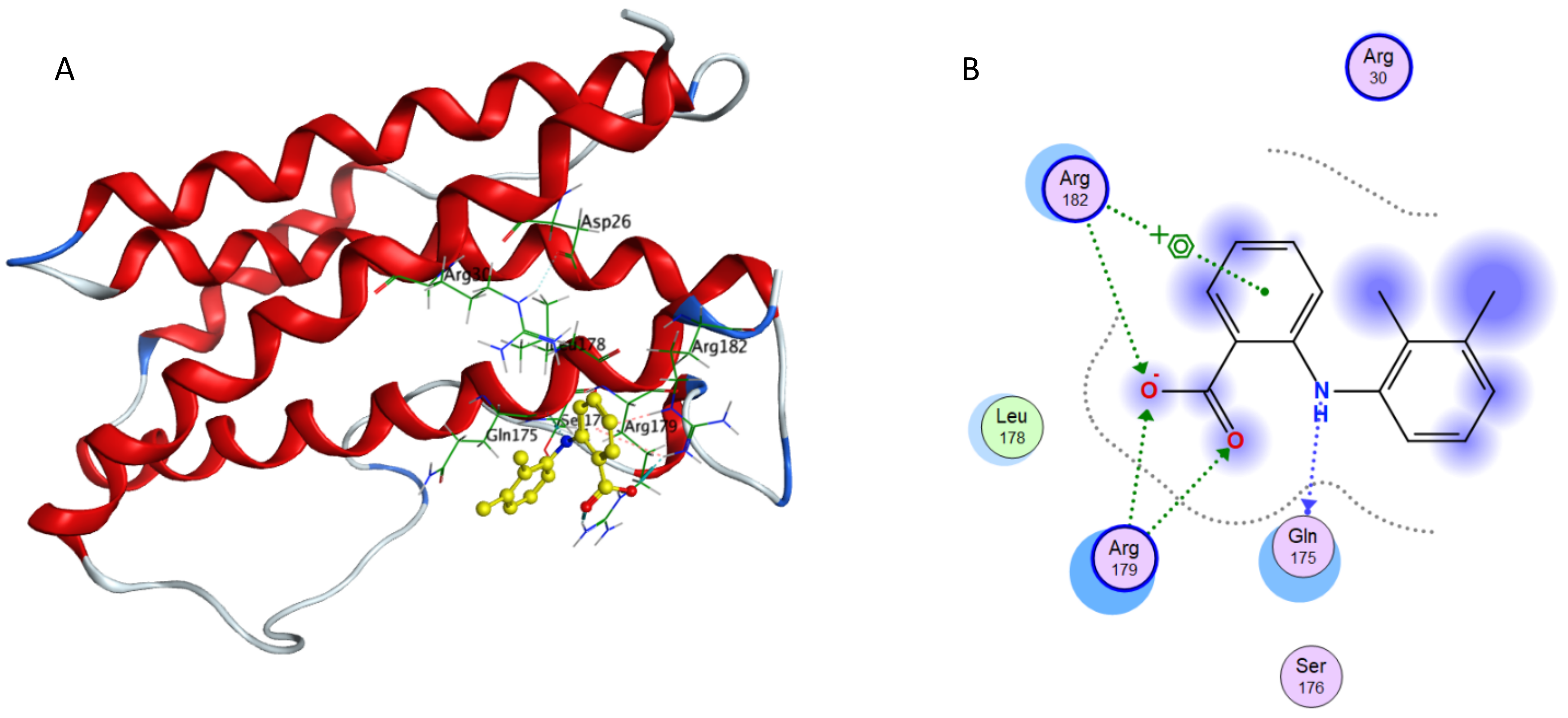

Supplement: Supplemental Information 2 — (A) Binding of mefenamic acid in the active site of IL-6; (A) Ligand interaction diagram of mefenamic acid in binding site of IL-6. [file peerj-08-9533-s002.png]

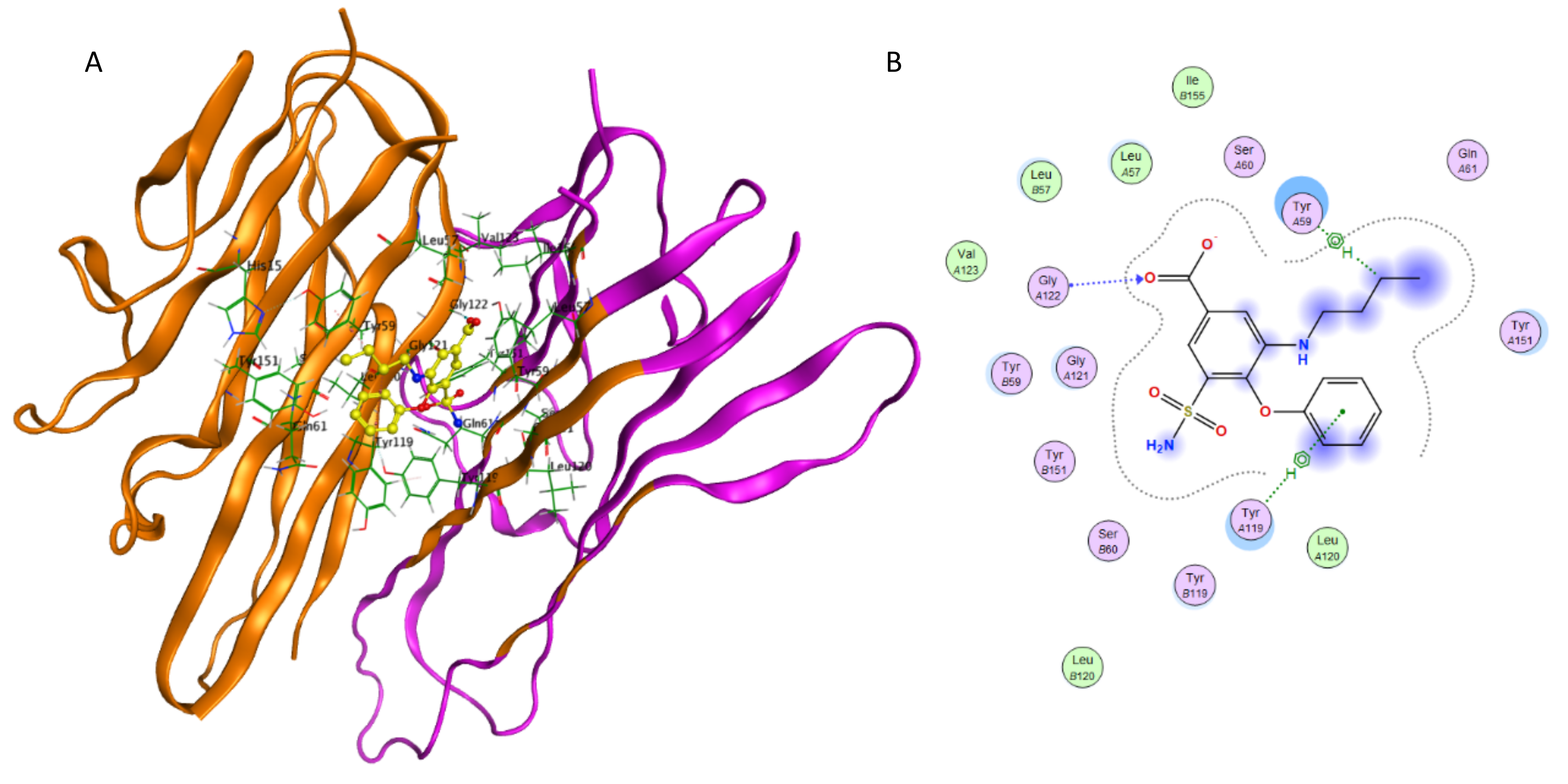

Supplement: Supplemental Information 3 — (A) Binding of bumetanide in the active site of TNF-α; (A) Ligand interaction diagram of bumetanide in binding site of TNF-α. [file peerj-08-9533-s003.png]

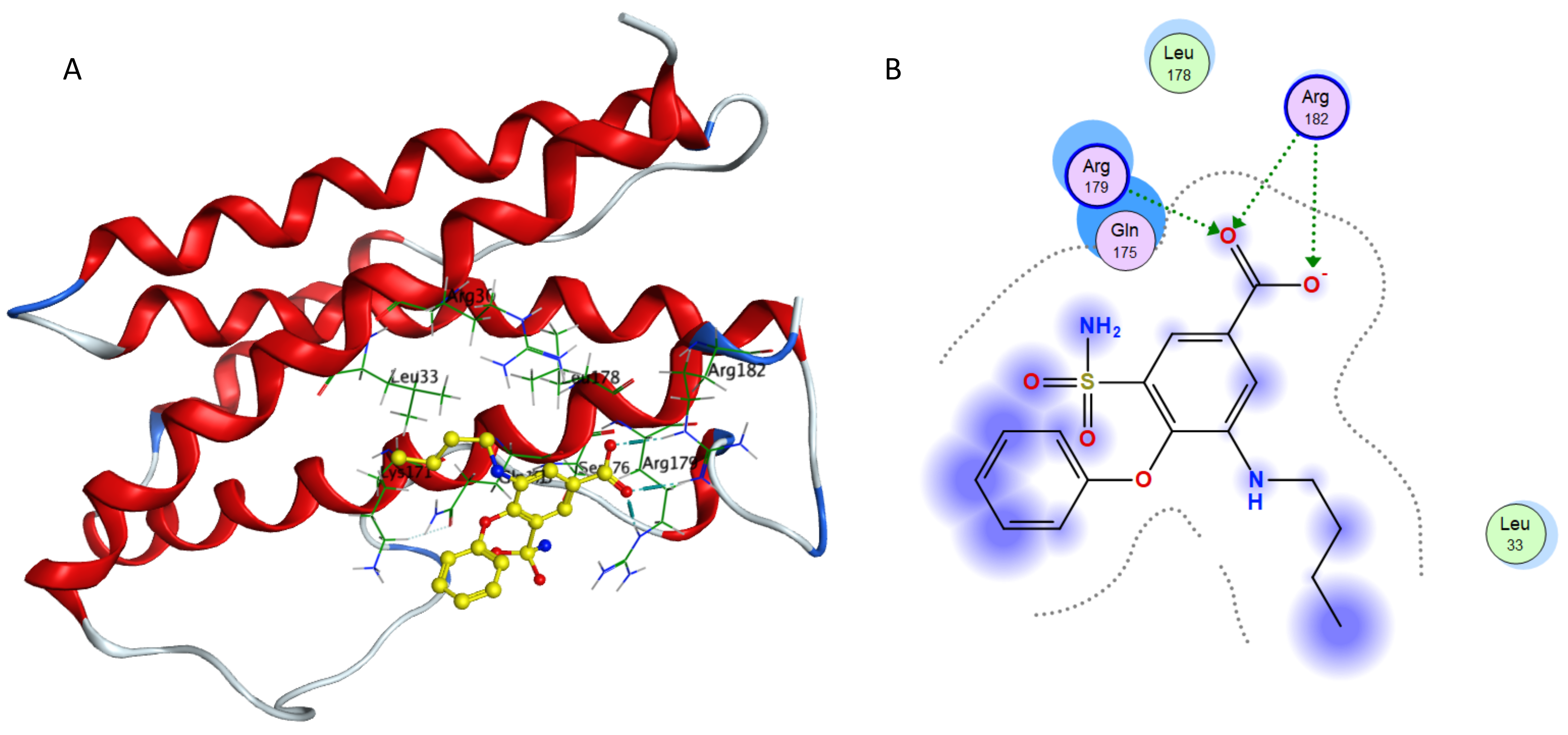

Supplement: Supplemental Information 4 — (A) Binding of bumetanide in the active site of IL-6; (A) Ligand interaction diagram of bumetanide in binding site of IL-6. [file peerj-08-9533-s004.png]

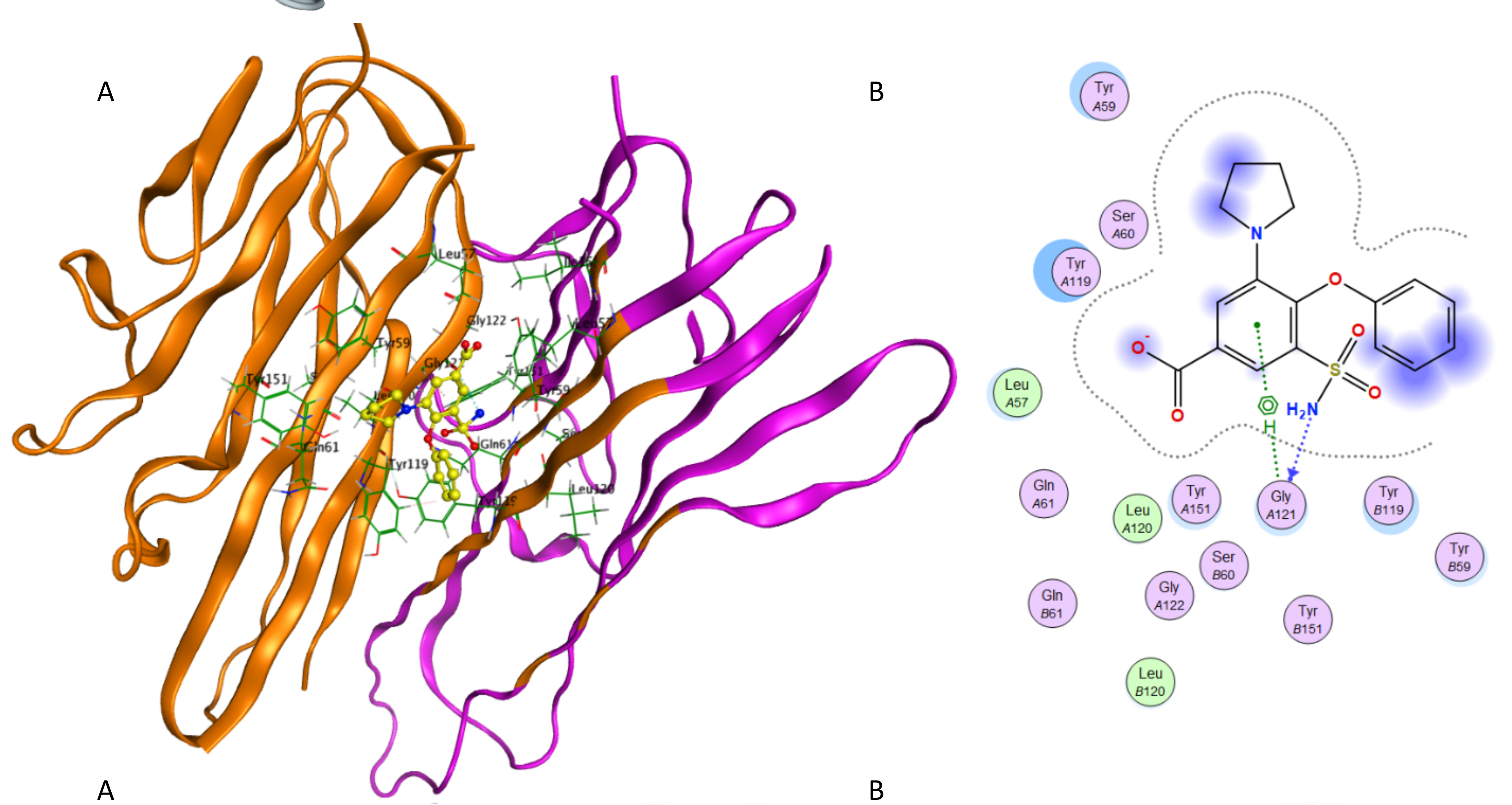

Supplement: Supplemental Information 5 — (A) Binding of piretanide in the active site of TNF-α; (A) Ligand interaction diagram of piretanide in binding site of TNF-α. [file peerj-08-9533-s005.png]

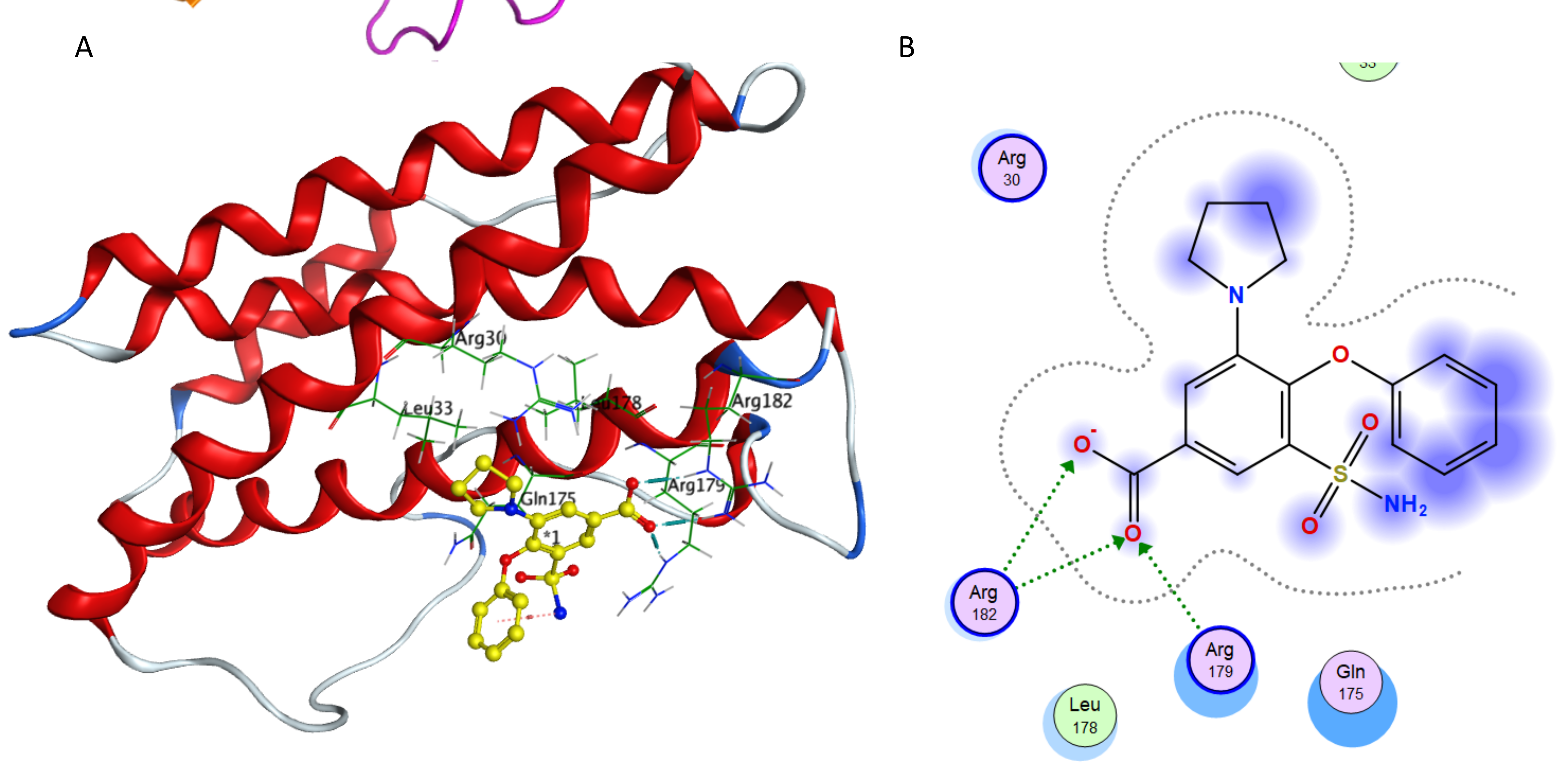

Supplement: Supplemental Information 6 — (A) Binding of piretanide in the active site of IL-6; (A) Ligand interaction diagram of piretanide in binding site of IL-6. [file peerj-08-9533-s006.png]

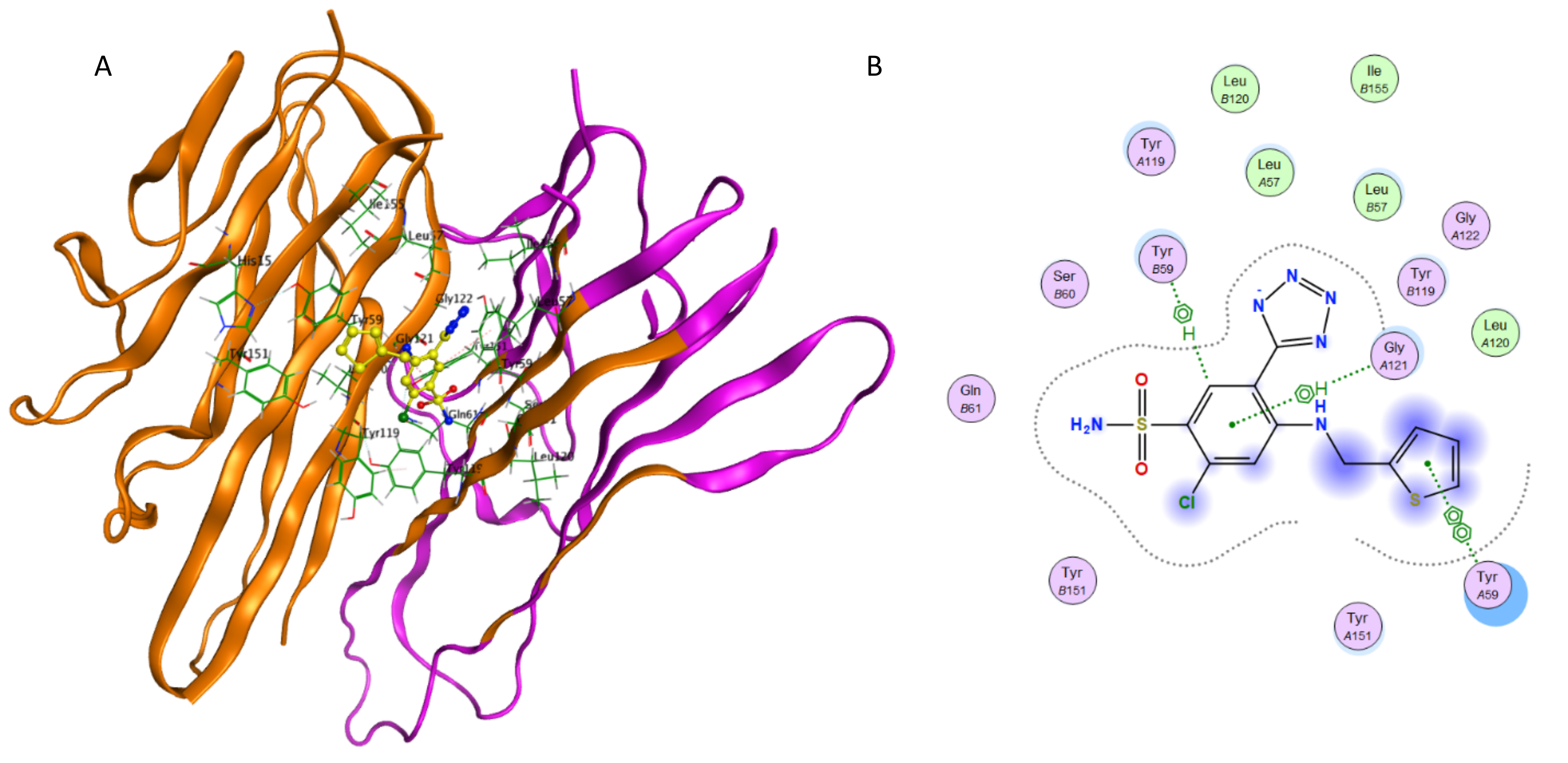

Supplement: Supplemental Information 7 — (A) Binding of azosemide in the active site of TNF-α; (A) Ligand interaction diagram of azosemide in binding site of TNF-α. [file peerj-08-9533-s007.png]

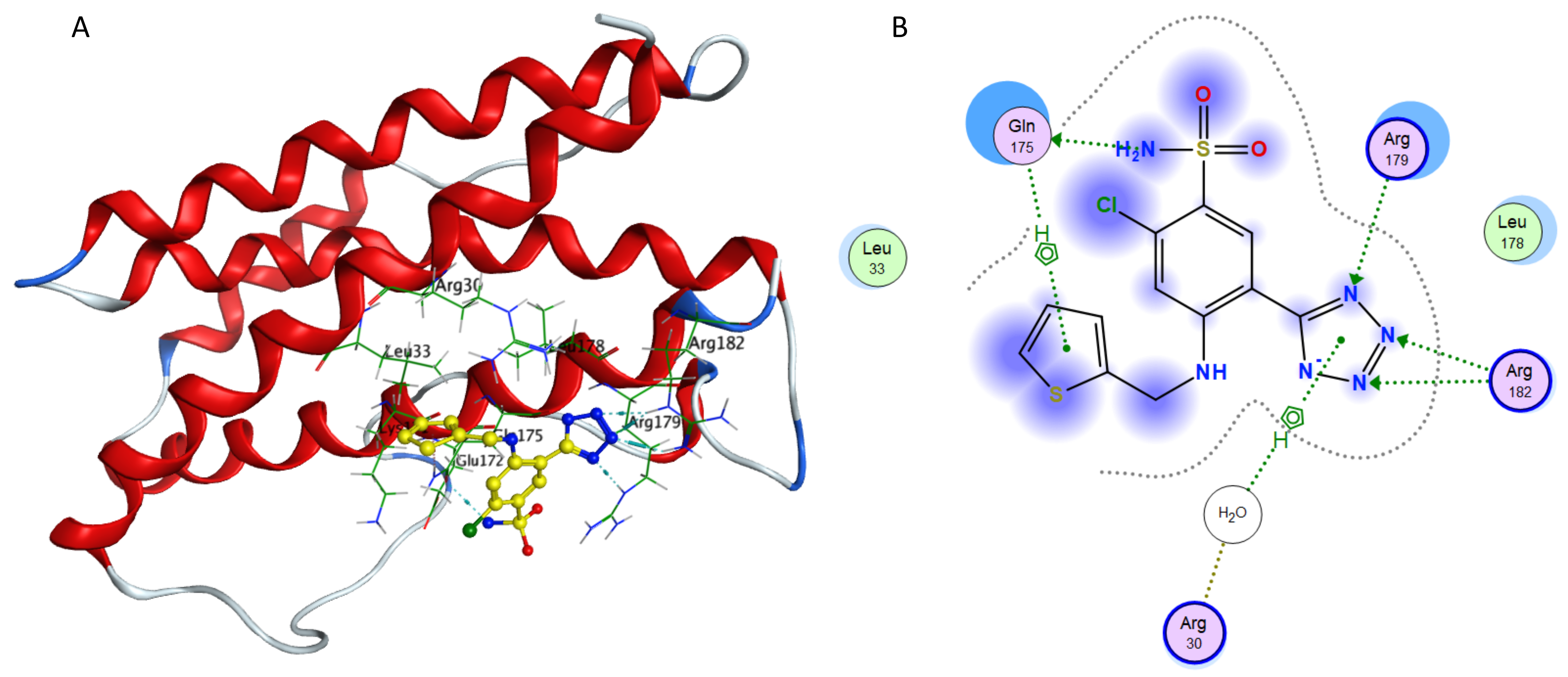

Supplement: Supplemental Information 8 — (A) Binding of azosemide in the active site of IL-6; (A) Ligand interaction diagram of azosemide in binding site of IL-6. [file peerj-08-9533-s008.png]

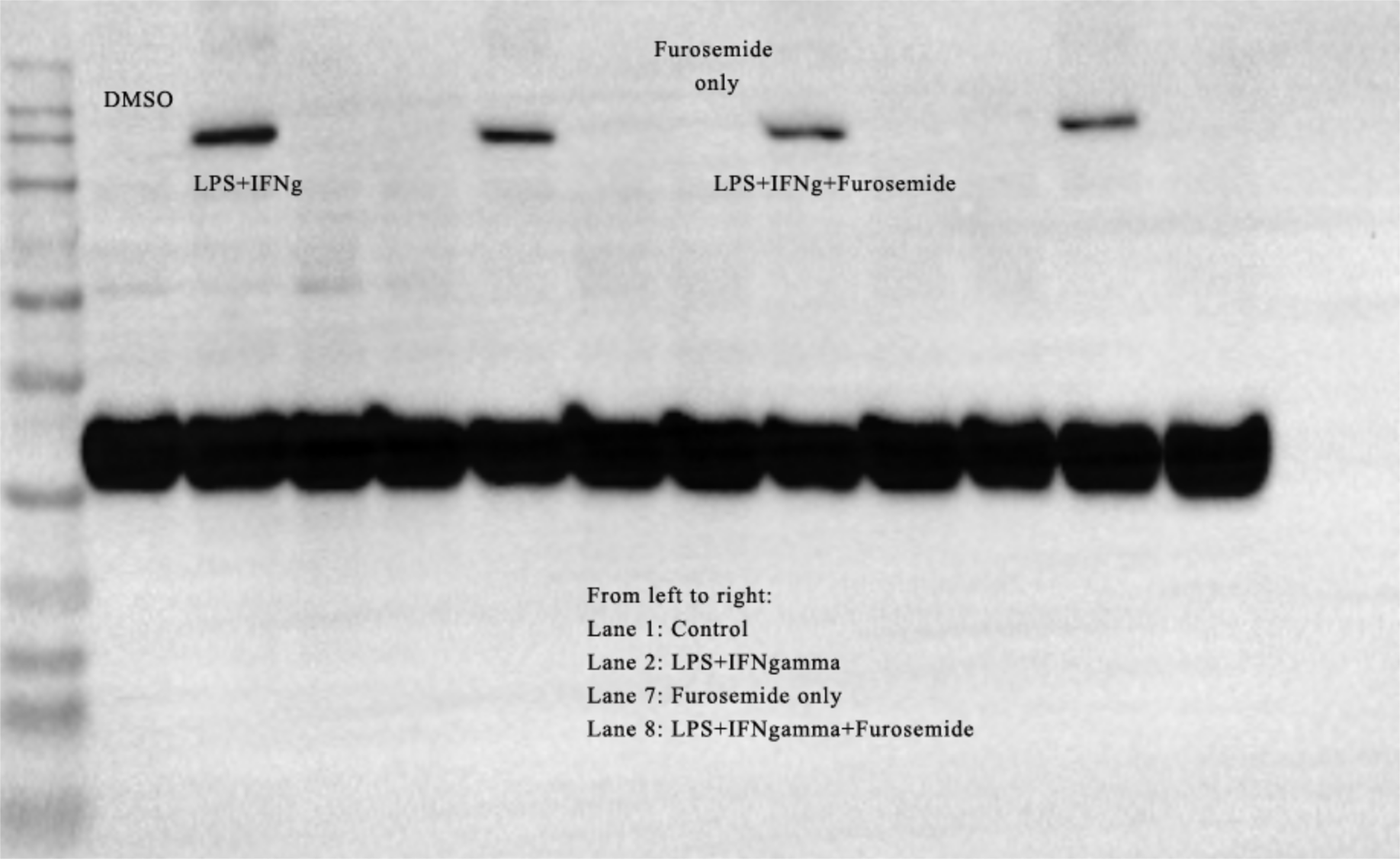

Supplement: Supplemental Information 9 [file peerj-08-9533-s009.png]

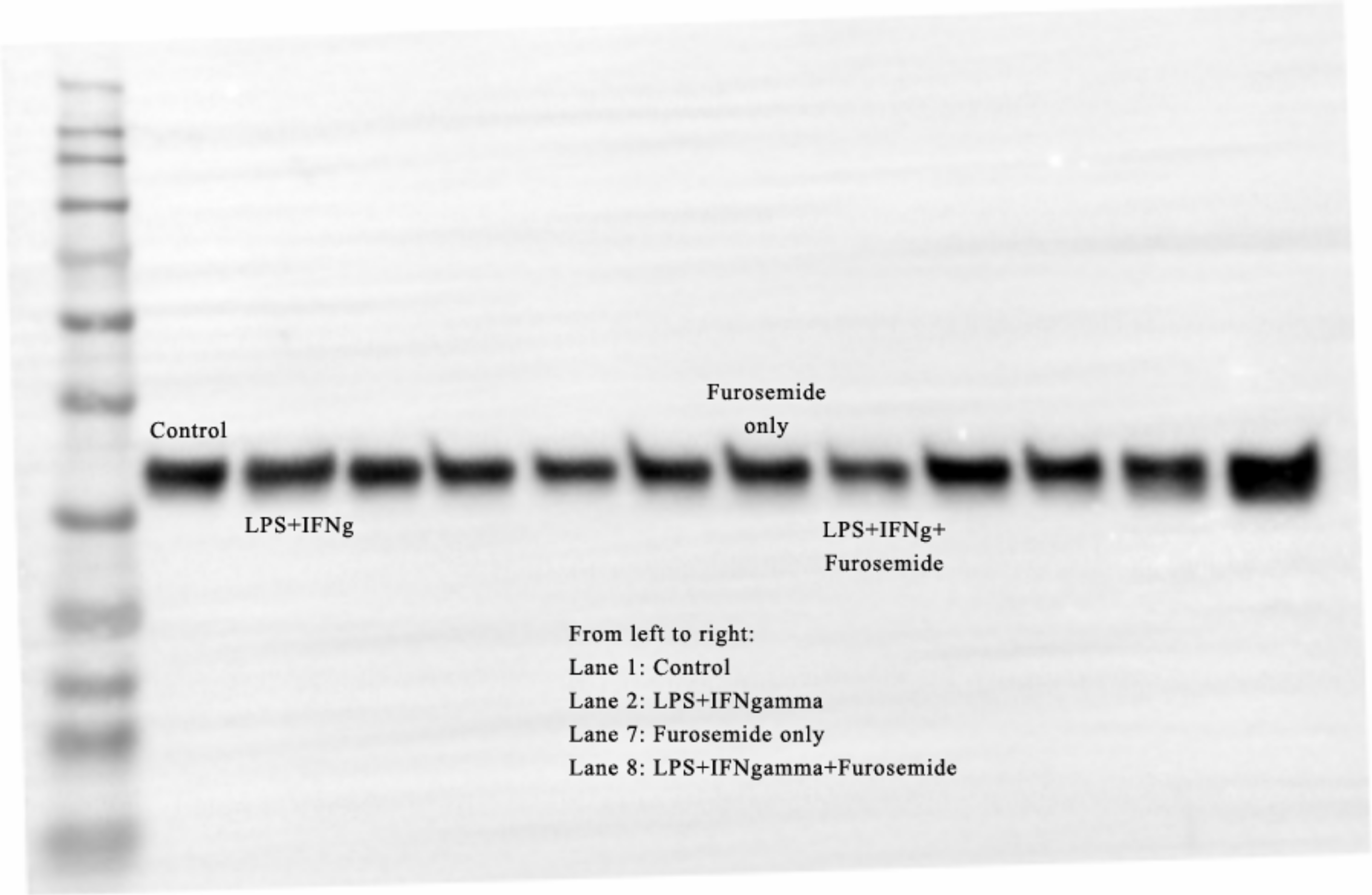

Supplement: Supplemental Information 10 [file peerj-08-9533-s010.png]

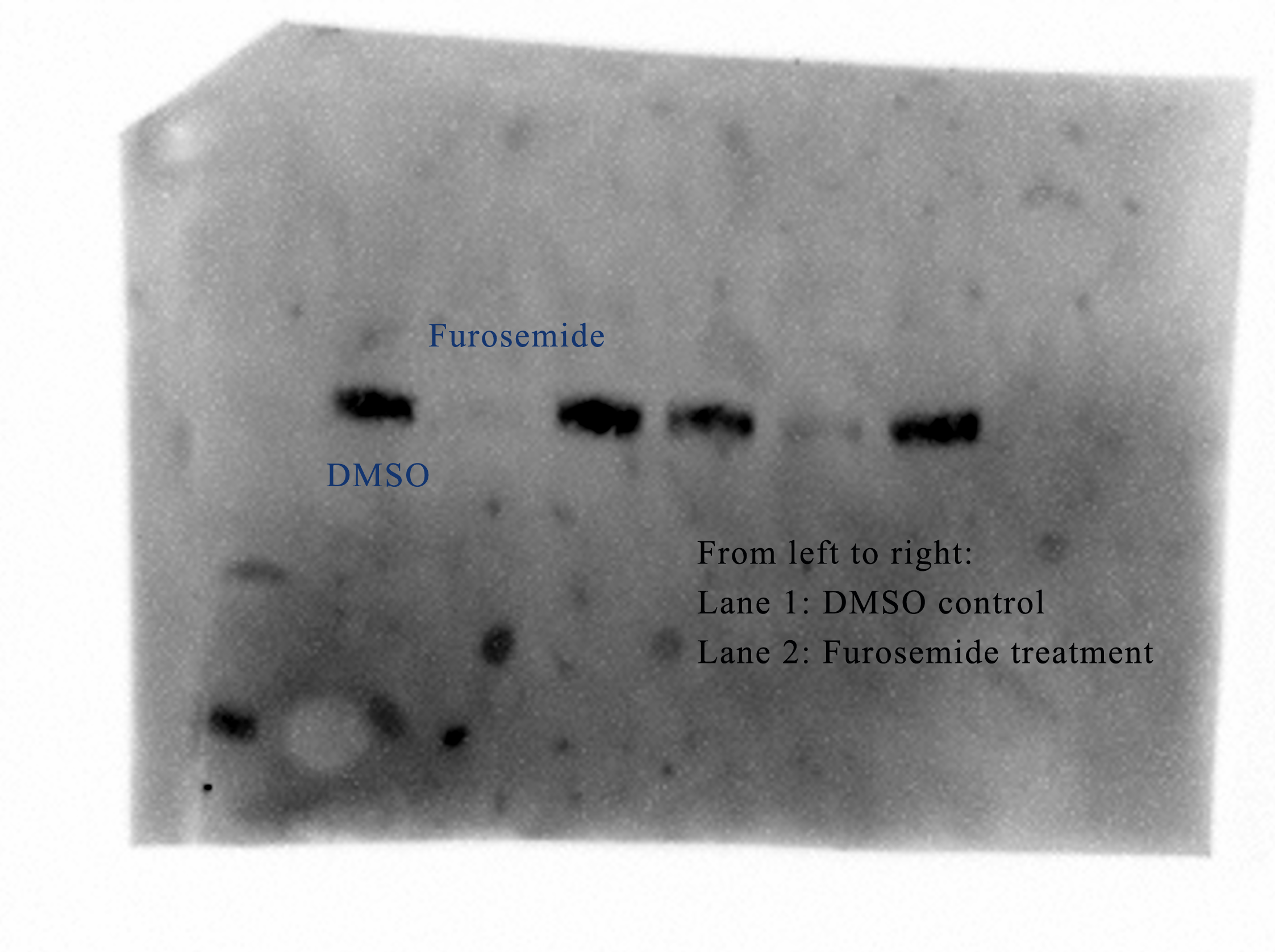

Supplement: Supplemental Information 11 [file peerj-08-9533-s011.png]

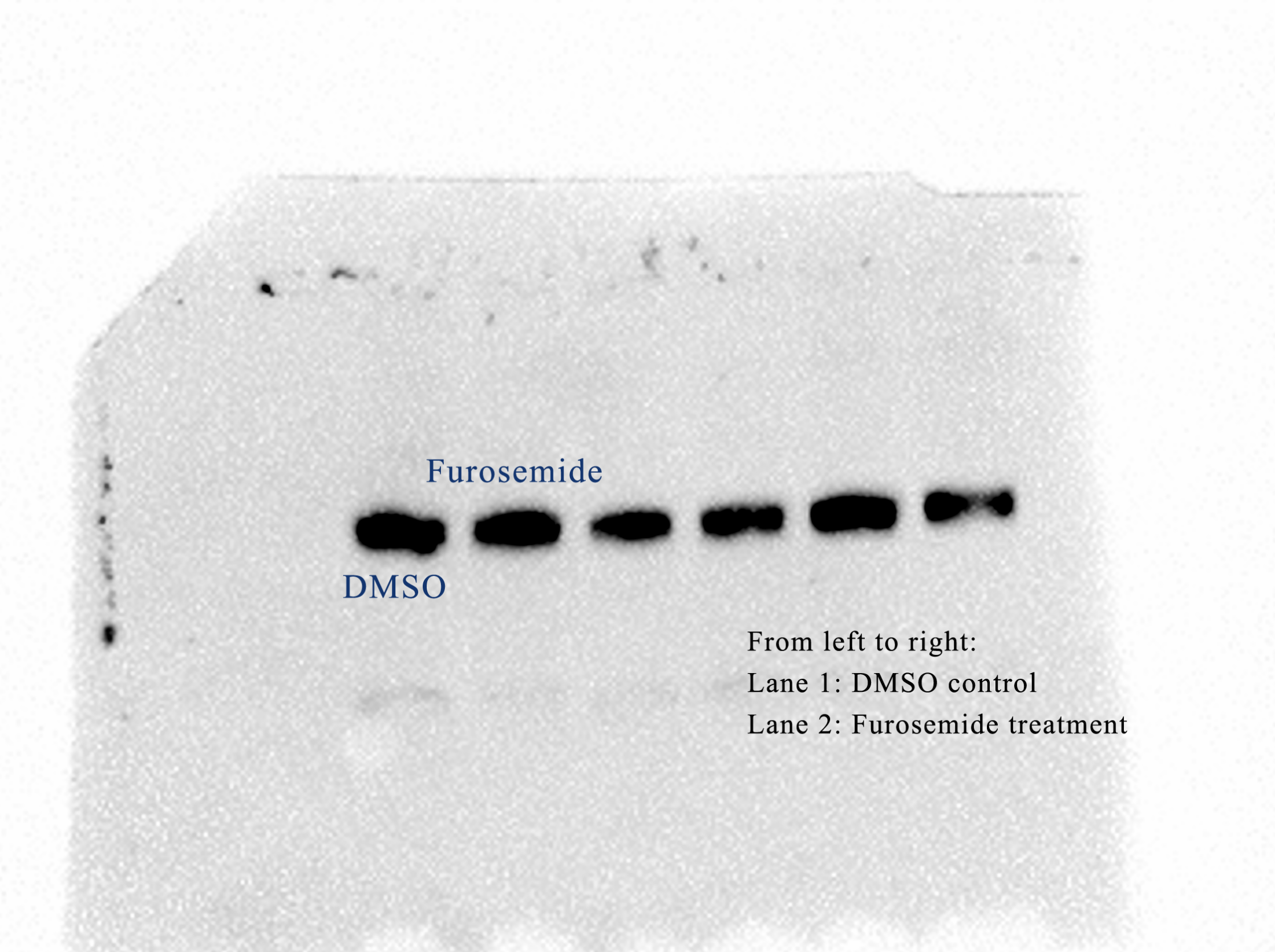

Supplement: Supplemental Information 12 [file peerj-08-9533-s012.png]
