# Supplementary material for: Small molecule therapeutics for COVID-19: repurposing of inhaled furosemide
Source: PeerJ. 2020 Jul 7;8:e9533. doi: 10.7717/peerj.9533 (PMC7350920; doi:10.7717/peerj.9533)
Supplement: Supplemental Information 13 [file peerj-08-9533-s013.docx]

**Table S1:** List of physiochemical descriptors used as initial screening test of the dataset.

| S.  No | Descriptor Name | Description | Software used to calculate value of descriptor |
| --- | --- | --- | --- |
| 1 | MW | Molecular weight | MOE^1^ |
| 2 | TPSA | Topological polar surface area | MOE |
| 3 | logP | log of octanol/water distribution coefficient | MOE |
| 4 | logD | Octanol/water distribution coefficient(MOE: pH = 7, CA: pH = 7.4) | MOE |
| 5 | pKa | Acidity (pH =7) | MOE |
| 6 | HBD | Hydrogen bond donor | MOE |
| 7 | HBA | Hydrogen bond acceptor | MOE |
| 8 | BBB Score | The blood-brain barrier Score^2^ | MOE |
| 9 | Ro5 violation | The Lipinski Violation Count^3^ | MOE |
| 10 | Mutagenicity | Toxicity and mutagenicity of the molecule | MOE |

^1^MOE corresponds to Molecular Operating Environment software

^2^For more information see: Gupta M, Lee HJ, Barden CJ, Weaver DF. The Blood-Brain Barrier (BBB) Score. J Med Chem 2019; 62(21):9824–36.

^3^For more information see: Lipinski CA, Lombardo F, Dominy BW, Feeney PJ. Experimental and computational approaches to estimate solubility and permeability in drug discovery and development settings. Adv Drug Deliv Rev 2001; 46(1-3):3–26.
