# Supplementary material for: Small molecule therapeutics for COVID-19: repurposing of inhaled furosemide
Source: PeerJ. 2020 Jul 7;8:e9533. doi: 10.7717/peerj.9533 (PMC7350920; doi:10.7717/peerj.9533)
Supplement: Supplemental Information 14 [file peerj-08-9533-s014.docx]

**Table S2:** List of promising drug candidates along with corresponding physiochemical descriptors.

| S.No. | Drug | HBA | HBD | BBB | logD | pKa | M | logP | TPSA | Weight |
| --- | --- | --- | --- | --- | --- | --- | --- | --- | --- | --- |
| 1 | Furosemide | 2 | 2 | 3.92 | 0.90 | 10.33 | 0 | 0.82 | 125.46 | 329.74 |
| 2 | 3-HAA | 1 | 2 | 4.26 | -0.17 | 9.68 | 0 | -0.66 | 86.38 | 152.13 |
| 3 | Mefenamic acid | 0 | 1 | 4.15 | 3.39 | 14.00 | 0 | 2.41 | 52.16 | 240.28 |
| 4 | Etacrynic acid | 2 | 0 | 4.10 | 3.96 | 14.00 | 0 | 2.27 | 66.43 | 302.13 |
| 5 | Torasemide | 4 | 3 | 3.64 | 2.34 | 14.00 | 0 | 2.53 | 100.19 | 348.43 |
| 6 | Trichlormethiazide | 4 | 3 | 3.61 | 0.74 | 9.36 | 1 | 0.82 | 118.36 | 380.66 |
| 7 | Chlorothiazide | 5 | 2 | 3.25 | 0.44 | 10.15 | 0 | 0.13 | 118.69 | 295.73 |
| 8 | Quinethazone | 3 | 3 | 3.82 | 0.42 | 10.27 | 0 | 0.88 | 101.29 | 289.74 |
| 9 | Meticrane | 4 | 1 | 4.18 | -0.17 | 10.18 | 0 | 0.36 | 94.30 | 275.35 |
| 10 | Metolazone | 3 | 2 | 4.50 | 2.63 | 10.26 | 0 | 2.71 | 92.50 | 365.84 |
| 11 | Methyclothiazide | 4 | 2 | 3.91 | 0.64 | 10.21 | 1 | 0.60 | 109.57 | 360.24 |
| 12 | Bumetanide | 2 | 2 | 3.87 | 1.94 | 10.15 | 0 | 1.70 | 121.55 | 363.41 |
| 13 | Methyclothiazide | 4 | 2 | 3.91 | 0.64 | 10.21 | 1 | 0.60 | 109.57 | 360.24 |
| 14 | Polythiazide | 4 | 2 | 3.97 | 1.04 | 10.21 | 0 | 1.66 | 109.57 | 439.89 |
| 15 | Cyclopenthiazide | 4 | 3 | 3.56 | 1.34 | 9.79 | 0 | 1.60 | 118.36 | 379.89 |
| 16 | Piretanide | 2 | 1 | 4.22 | 1.31 | 10.15 | 0 | 1.09 | 112.76 | 361.40 |
| 17 | Amiloride | 3 | 2 | 2.86 | -4.05 | 11.14 | 1 | -2.90 | 158.53 | 230.64 |
| 18 | Methazolamide | 4 | 2 | 3.01 | -1.69 | 9.87 | 0 | -1.36 | 104.86 | 238.29 |
| 19 | Hydroflumethiazide | 4 | 3 | 3.46 | -0.58 | 9.48 | 0 | 0.33 | 118.36 | 331.29 |
| 20 | Diazoxide | 3 | 1 | 4.29 | 2.16 | 14.00 | 0 | 1.87 | 58.53 | 230.68 |
| 21 | Indapamide | 3 | 2 | 4.51 | 2.52 | 10.16 | 0 | 2.08 | 92.50 | 365.84 |
| 22 | Bendroflumethiazide | 4 | 3 | 3.75 | 1.34 | 9.53 | 0 | 1.94 | 118.36 | 421.42 |
| 23 | Benzthiazide | 5 | 2 | 3.76 | 2.68 | 10.15 | 0 | 2.70 | 118.69 | 431.95 |
| 24 | Azosemide | 2 | 2 | 4.04 | 0.24 | 10.37 | 0 | 1.74 | 110.86 | 369.84 |
| 25 | Clopamide | 4 | 2 | 4.11 | 1.20 | 10.17 | 0 | 1.90 | 92.50 | 345.85 |

TPSA, HBA, HBD, BBB, M corresponds to topological polar surface area, hydrogen bond acceptor, hydrogen bond donor, blood-brain barrier score and mutagenicity respectively. For details on BBB score see: Gupta M, Lee HJ, Barden CJ, Weaver DF. The Blood-Brain Barrier (BBB) Score. J Med Chem 2019; 62(21):9824–36.
