# Supplementary material for: Small molecule therapeutics for COVID-19: repurposing of inhaled furosemide
Source: PeerJ. 2020 Jul 7;8:e9533. doi: 10.7717/peerj.9533 (PMC7350920; doi:10.7717/peerj.9533)
Supplement: Supplemental Information 15 [file peerj-08-9533-s015.docx]

| Drug | Docking Score 'S' with IL-6 | Docking Score 'S' with TNF-α |
| --- | --- | --- |
| Furosemide | -5.13 | -6.09 |
| 3-HAA | -4.62 | -4.49 |
| Mefenamic acid | -5.39 | -5.72 |
| Etacrynic acid | -5.21 | -5.47 |
| Torasemide | -5.70 | -6.64 |
| Trichlormethiazide | -4.76 | -5.72 |
| Chlorothiazide | -4.69 | -5.44 |
| Quinethazone | -5.25 | -5.90 |
| Meticrane | -4.74 | -5.49 |
| Metolazone | -5.17 | -5.79 |
| Methyclothiazide | -5.32 | -5.77 |
| Bumetanide | -5.58 | -6.63 |
| Methyclothiazide | -5.20 | -5.93 |
| Polythiazide | -5.58 | -6.80 |
| Cyclopenthiazide | -4.98 | -6.28 |
| Piretanide | -5.30 | -6.71 |
| Amiloride | -4.65 | -4.94 |
| Methazolamide | -4.85 | -5.33 |
| Hydroflumethiazide | -4.58 | -5.71 |
| Diazoxide | -4.69 | -5.21 |
| Indapamide | -5.37 | -5.89 |
| Bendroflumethiazide | -5.11 | -6.45 |
| Benzthiazide | -5.46 | -6.38 |
| Azosemide | -5.75 | -6.38 |
| Clopamide | -5.46 | -6.38 |

**Table S3:** Docking Score ‘S’ of promising drug candidates with IL-6 and TNF-α.
